# Assessing the health status and mortality of older people over 65 with HIV

**DOI:** 10.1371/journal.pone.0241833

**Published:** 2020-11-05

**Authors:** Gina Turrini, Stephanie S. Chan, Pamela W. Klein, Stacy M. Cohen, Antigone Dempsey, Heather Hauck, Laura W. Cheever, Andre R. Chappel

**Affiliations:** 1 U.S. Department of Health and Human Services, Office of the Assistant Secretary for Planning and Evaluation, Office of Health Policy, Washington, DC, United States of America; 2 Division of Policy and Development, U.S. Department of Health and Human Services, Health Resources and Services Administration, HIV/AIDS Bureau, Rockville, Maryland, United States of America; Indiana University Purdue University at Indianapolis, UNITED STATES

## Abstract

**Background:**

Nearly half of people with HIV in the United States are 50 years or older, and this proportion is growing. Between 2012 and 2016, the largest percent increase in the prevalence rate of HIV was among people aged 65 and older, the eligibility age for Medicare coverage for individuals without a disability or other qualifying condition. Previous work suggests that older people with HIV may have higher rates of chronic conditions and develop them more rapidly than older people who do not have HIV. This study compared the health status of older people with HIV with the older US population not living with HIV by comparing: (1) mortality; (2) prevalence of certain conditions, and (3) incidence of these conditions with increasing age.

**Methods and findings:**

We used a sample of Medicare beneficiaries aged 65 and older from the Medicare Master Beneficiary Summary File for the years 2011 to 2016, including 100% of individuals with HIV (N = 43,708), as well as a random 1% sample of individuals without diagnosed HIV (N = 1,029,518). We conducted a survival analysis using a Cox proportional hazards model to assess mortality and to determine the need to adjust for differential mortality in our analyses of the incidence of certain chronic conditions. These results showed that Medicare beneficiaries living with HIV have a significantly higher hazard of mortality compared to older people without diagnosed HIV (3.6 times the hazard). We examined the prevalence of these conditions using logistic regression analysis and found that people with HIV have a statistically significant higher odds of depression, chronic kidney disease, chronic obstructive pulmonary disease (COPD), osteoporosis, hypertension, ischemic heart disease, diabetes, chronic hepatitis, end-stage liver disease, lung cancer, and colorectal cancer. To look at the rate at which older people are diagnosed with conditions as they age, we used a Fine-Gray competing risk model and showed that for individuals without diagnosis of a given condition at age 65, the future incidence of that condition over the remaining study period was higher for people with HIV even after adjusting for differential hazard of mortality and for other demographic characteristics. Many of these results also varied by personal characteristics including Medicaid dual enrollment, sex, and race and ethnicity, as well as by condition.

**Conclusions:**

Increasing access to care and improving health outcomes for people with HIV is a critical goal of the National HIV/AIDS Strategy 2020. It is important for clinicians and policymakers to be aware that despite significant advances in the treatment and care of people with HIV, older people with HIV have a higher odds of having multiple chronic conditions at any point in time, a higher incidence of new diagnoses of these conditions over time, and a higher hazard of mortality than Medicare beneficiaries without HIV.

## Introduction

Treatments for HIV have improved significantly over the past 24 years since combination antiretroviral therapy was first introduced in 1996. HIV is now a manageable, chronic condition and individuals retained on anti-retroviral therapy have much longer life expectancies than in the past, which is contributing to an ongoing shift in the age distribution of people with HIV [1–5]. This trend, seen in the US and elsewhere [6], toward an aging of the population with HIV raises important questions regarding the health care needs of older people with HIV, how they may differ from those of older people without HIV, and how best to address those needs.

While treatments have allowed people with HIV to live longer and have decreased the likelihood of AIDS-defining illnesses, other health conditions such as various non-communicable diseases appear to be becoming more common among older people with HIV. There are a number of potential reasons why older people with HIV may have different baseline health statuses or trajectories than older people without HIV, and the reasons likely differ by condition. First, HIV itself leads to chronic inflammation, which in turn is associated with systemic chronic immune activation and a higher likelihood of a number of diseases [7–10]. Second, the long-term toxicity of antiretroviral therapies (ARTs) may directly affect the body and may also interact with other medications [11]. Although newer treatment regimens cause fewer side effects than those used in the past, long-term use of ART is associated with increased risk of heart disease and heart attack; use of ART in general may lead to a number of health problems, including liver toxicity, pancreatitis, neuropathy, and impaired glucose metabolism [12, 13]. A third issue is that older people who are newly diagnosed with HIV tend to have more advanced disease due to delayed diagnosis, leading to more advanced immunodeficiency and an increased likelihood of blunted immune response to ART due to immune senescence [14]. This may be partly the result of barriers to early testing and diagnosis for older individuals, such as a lack of awareness of how risk factors may be different for older adults compared to younger individuals, as well as an underestimation of the risk of HIV infection at older ages both by patients and providers [14–18]. Delays in HIV diagnosis are associated with negative health outcomes, decreased life expectancy, and increased HIV transmission [19]. Finally, rates of HIV infection are higher in certain populations that tend to have poorer long-term health outcomes generally, including among men who have sex with men, transgender women, racial and ethnic minorities, and people experiencing poverty [20, 21]. Recently, the prevalence of HIV among people living in rural areas has been increasing, and these individuals are more likely to have delayed diagnosis and to face additional barriers to accessing health care, in general, and from providers with experience treating HIV, in particular [22]. In addition, a range of other adverse health behaviors, such as smoking, are disproportionately common among people with HIV [23, 24].

Previous research and anecdotal evidence from providers have raised concerns that, despite often having high rates of adherence and viral suppression [25, 26], older people with HIV may have a higher prevalence of chronic conditions overall and may develop new conditions at an earlier age. However, currently little is known about the health trajectory of older people with HIV as they age given the limitations of methods and data used in previous studies, particularly the use of cross-sectional data to examine point prevalence of various outcomes and the use of smaller or selected samples of people with HIV (for instance, a limited number of clinics from The HIV Outpatient Study [27], individuals in Medicare from 2006–2009 [28], Medicaid enrollees with HIV are not older adults [29]). Cross-sectional analyses that simply compare the average age of onset of a condition or disease can potentially lead to incorrectly concluding that there is evidence of premature aging if population age structure differences are not accounted for. Given that there are fewer older people with HIV than there are without HIV, the unadjusted mean age of onset may be lower even if the risk or incidence at a given age is actually the same [30]. Cross-sectional analysis also limits the ability to account for possible selection effects from differences in mortality rates. The few studies that try to use cross-sectional data to look at incidence and premature aging have mixed findings [31]. Without following patients over time, it is difficult to distinguish between the effects of “premature” aging, where diagnoses of conditions occur at earlier ages, or “accentuated” aging, where people with HIV have higher prevalence rates of certain conditions than people without diagnosed HIV at every age but there is no change in the average age of onset [32]. The ability to distinguish between premature and accentuated aging can enable health providers and programs serving older people with HIV to better care for and prepare for the needs of this population, especially if the accumulation of heath conditions of older people with HIV follow a trajectory that involves more conditions, higher severity, and a faster timeline. Together, these previous studies suggest that even among older people with HIV on antiretroviral therapy, there are higher rates of cardiovascular disease, heart disease, chronic hepatitis, chronic obstructive pulmonary disease (COPD), renal disorders, osteoporosis, and non-AIDS related cancers (e.g. lung and colorectal cancer), among other conditions, compared to those without an HIV diagnosis. Likewise, these patients are more likely to have multiple comorbidities and polypharmacy compared to older people without diagnosed HIV [11, 12, 18, 27–29, 33–42] for a selection of examples].

We were able to find one set of published analyses of a large, representative cohort, the Veterans Aging Cohort Studies, which followed a large number of veterans with and without HIV over time, but these results may not be generalizable to other populations (the sample was almost entirely male, for instance, and younger on average than our population of interest; see [43, 44] for two examples from the Veterans Aging Cohort Studies). From the Veterans Aging Cohort Study, there was a small but statistically significant increased association between general physical decline and age for older people with HIV compared to older people without HIV [43], and a statistically significantly larger association between age and diabetes, vascular disease, and liver disease for older people with HIV compared to older people without HIV [44]. Another Veterans Aging Cohort Study analysis found that adjusted mean age of myocardial infarction and non-AIDS defining cancer were not significantly different for people with HIV and people without HIV, but HIV-infected adults were diagnosed with end-stage renal disease, on average, 5.5 months younger than people without HIV [33].

This current study builds on the existing evidence base by using longitudinal data from the general population to compare the health status of older people with HIV with the older US population not living with HIV by comparing: (1) mortality; (2) prevalence of several important, often co-occurring, conditions; and (3) incidence of these conditions as individuals age. This study used the Medicare Master Beneficiary Summary File (MBSF) from 2011 to 2016, which allowed us to track the health status of a large, nationally representative sample of older people with HIV, aged 65 and older, while accounting for individual characteristics and survival trends. This also allowed us to extend our analyses to look at populations that may be differentially affected by aging with HIV, including by urban/rural residence, Medicaid enrollment, sex, and race/ethnicity. As far as we are aware, this is the first study to use a nationally representative cohort of Medicare enrollees over time to look at aging with HIV, and in particular, to account for differential mortality.

## Methods

### Data and samples

The data for this project came from the Centers for Medicare & Medicaid Services (CMS) Master Beneficiary Summary File (MBSF), which contains data on all individuals enrolled in Medicare in a given calendar year. Citizens and permanent residents of the United States are generally eligible for Medicare at age 65 or if they have end-stage renal disease or have a disability and have accumulated a sufficient amount of work history individually or through a spouse or parent to qualify for Social Security Disability Insurance (SSDI) or Social Security Retirement Benefits or Railroad Retirement benefits or a railroad disability annuity. We used a random 1% sample of beneficiaries who never had an HIV diagnosis and 100% of the individuals who ever had an HIV diagnosis in the MBSF files from 2011 to 2016 and then compared these two groups of individuals as long as they were alive for those years. We chose to limit our analysis to 2011 to 2016 because of concern about a CMS rule change noted in Pub 100–04 Medicare Claims Processing (Transmittal 2028) beginning in January 2011, which increased the number of diagnosis codes that could be recorded on a claim from 9 to 25 (potentially making people artificially appear sicker than they were before the change). Individuals could enter the analytic sample in any year after age 65; we observed beneficiaries annually up to six years (five years on average). For all mortality and incidence analyses, our unit of time was therefore the calendar year. Although we were able to analyze outcomes for the entire population of people with HIV, we used a 1% sample of beneficiaries without a diagnosis of HIV for comparison due to computing limitations and because sensitivity analyses showed very similar results that did not change our findings with a 5% sample. Our sample included 1,073,226 individuals aged 65 and older, of whom 43,708 had an HIV diagnosis. As expected, individuals with an HIV diagnosis (49,561) accounted for a small percentage (0.1%) of the entire population of Medicare fee-for-service beneficiaries (50,360,645) in 2016. We excluded those younger than age 65 who appeared in our data (namely, those who qualified for Medicare coverage based on disability or because they had end-stage renal disease and not because of age). The MBSF files contained beneficiary-year level data on demographic and geographic characteristics, whether an individual was enrolled in Medicaid, diagnosis flags for a number of conditions (including diagnosis of HIV or AIDS), and date of death (if applicable). The diagnosis flags were hierarchical condition category (HCC) codes developed by CMS, which used International Classification of Diseases (ICD) codes from medical claims and mapped them to particular disease groups (for instance, diabetes or chronic kidney disease) [45].

### Statistical methods

We conducted three sets of regression analyses to compare: (1) mortality; (2) prevalence of selected chronic conditions; and (3) incidence of these conditions among older people living with and those without an HIV diagnosis. In all models, our primary explanatory variable of interest was the HIV diagnosis indicator (the comparison group is older adults without an HIV diagnosis ever during the study period). For prevalence and incidence comparisons, we selected a set of health conditions hypothesized to be associated with HIV infection as well as others that are generally associated with older ages (many, such as cardiovascular disease, are associated with both). We analyzed the following 11 conditions as outcome variables in separate regressions: depression, hypertension, chronic kidney disease, chronic obstructive pulmonary disease (COPD), osteoporosis, heart disease, colorectal cancer, lung cancer, diabetes, chronic hepatitis, and end-stage liver disease. For people with HIV, these were comorbid conditions to their HIV, while for people without HIV, these may have been their only chronic condition.

In our first set of models, we used survival analyses to assess whether older people with HIV have a higher hazard of mortality over time than older individuals without HIV. We did this both to analyze mortality as an outcome, and to assess the extent to which selective mortality may be a concern for subsequent chronic condition models, which was the focus of our analysis. To do this, we used a Cox proportional hazards model to compare the mortality hazard between older people with HIV and older people without diagnosed HIV as they age. For this set of models, we further restricted our overall sample for this analysis to individuals we observed at age 65 (between 2011 and 2016) and were observed annually or until they died. This was done because we were interested in comparing the health status of individuals beginning when they first age into Medicare. The Cox proportional hazard models included covariates for sex, race and ethnicity (non-Hispanic Black, non-Hispanic White, non-Hispanic Asian, and Hispanic), dual enrollment in Medicaid and Medicare (a proxy for low-income status), rural (or non-core based statistical area [46]) or urban residence, and indicator variables for the year they turned 65 (to control for temporal changes in factors such as treatment and cohort composition). Additionally, interaction terms with exposure time (years since 65) were included for the indicators for HIV diagnosis and for non-Hispanic Black designation, based on failures of the checks for the proportional hazards assumption for those covariates conducted using Schoenfeld residuals [47]. To look at population subgroups of interest, additional extended models included interactions between HIV diagnosis and individual characteristics including Medicaid dual enrollment, rural or urban location, sex, and race or ethnicity. For all results, hazard ratios (HR) and 95% confidence intervals (CI) were reported.

One potential limitation of this age restriction is that, given our six years of data, we were only looking at the ages of 65 to 71 (among the people with HIV in our sample who were at least 65, approximately 39% of them are older than 71). To address whether the results would be significantly changed if we were to look at a wider age range, we ran several robustness checks (S1 Table). The first robustness check analyzed individuals beginning when they turned 75 (rather than 65). The second robustness check included individuals at all ages but adjusted for age. The results of both of these robustness checks were extremely similar to our primary results. The main specification for our mortality and incidence analyses also removed anyone who was newly diagnosed with HIV after the age of 65 (12,566 Medicare beneficiaries, approximately 1% of our sample). Although this was a small number of individuals, they may be quite different from the vast majority who are diagnosed at younger ages and who have been living with the diagnosis and potentially in treatment for many years. For the third robustness check we ran these analyses with them included and this did not substantially change our results (results in S1 Table). In addition, there may be concern that HIV diagnosis may be associated with health conditions which themselves are associated with mortality (as we will illustrate with the second set of analyses on prevalence). We considered matching or including these conditions as covariates but ultimately chose not to in our preferred specification because of the concern that HIV itself may be a contributing factor for many of those conditions, so including (or matching) based on them would be capturing some of the effects of HIV as well. However, in the fourth robustness check in S1 Table, we show that there is still a significant, although somewhat attenuated, relationship between HIV diagnosis and mortality even if we include the other conditions as covariates, and also if we use an inverse probability weighted model. We did not use inverse probability weighting or other similar propensity score methods given the limited number of other demographic and personal characteristics available in the data. Another possibility was to match on our small set of demographic characteristics to create a control group that appears more similar in terms of observed characteristics to the population of older people with HIV on those dimensions. However, this would not necessarily reduce confounding from unobserved characteristics compared to using the full sample and adjusting for observed characteristics [48], and therefore we chose not to employ a matching method.

In our second set of models, we investigated whether older people with HIV have a higher prevalence of each of the previously mentioned conditions at any given age, not adjusting for differential mortality. For each outcome, we ran a logistic regression model using 2016 data only in order to understand the prevalence at a given point in time (odds ratios [OR] and 95% CI are reported). The models were also run for other years and the results did not substantially change; for simplicity, only 2016 is presented in the main table although other years are shown in S2 Table. The models also controlled for age, sex, race and ethnicity, dual enrollment in Medicaid, rural or urban residence, as well as state of residence. Whereas the primary set of models include everyone, we were also interested in whether any observed patterns varied by select stratification variables: rural residence, Medicaid dual enrollment, sex, and race and ethnicity. To look at this question, we ran similar models but also included interactions between each covariate and the stratification variable (fully saturated models). The main specification for the prevalence models include everyone age 65 and older and adjusts for age, whereas the survival analysis (and incidence analysis discussed below) uses a more restricted sample of individuals who we observed as they enter Medicare. We tested whether restricting the prevalence sample to individuals who are included in the mortality and incidence regressions changed the results, and the results were very similar (S2 Table). While some researchers have a preference for risk ratios over ORs, particularly for more common events when ORs and risk ratios are more likely to diverge, we chose to use a logit and report ORs which are informative, even for common outcomes, if interpreted appropriately. In addition, there are some researchers who argue that there are methodological reasons for preferring ORs, and that risk ratios may actually be worse in cases where outcomes are common or the underlying risk in a population may not be homogeneous [49].

Given that we found a higher mortality rate for older people with HIV in the first set of models, for our third set of models, we used a competing risk model based on Fine and Gray [50] to analyze whether the incidence of new conditions occurs more rapidly for people with HIV, but with a competing risk that an individual may die before developing the condition. A standard time to event model analyzing incidence would treat individuals who die as if they would otherwise have the same risk of developing the condition as the people who remain alive. However, this assumption seems unrealistic if individuals who die are less healthy overall. Given their worse health status, it seems reasonable to hypothesize that they would be more likely to develop additional conditions. A competing risk model enables us to account for this situation. Similar to the survival models, we restricted the sample to individuals who we observed when they turned 65. The competing risk models therefore analyzed the time to diagnosis or death for individuals who have not yet been diagnosed with a particular condition of interest as of age 65. Individuals enter the analysis at age 65 and exit because of death or because we reach the end of the observation period. Our basic model included an indicator for diagnosis of HIV infection as well as the same set of demographic characteristics used in the mortality models. To look at population subgroups of interest in extended models, we included interactions between HIV diagnosis and personal characteristics of interest including Medicaid dual enrollment, rural and urban location, sex, and race and ethnicity (as we did in the mortality analysis). All results are reported as subhazard ratios with 95% CI.

Due to the relatively large number of outcomes of interest in the analyses of prevalence and incidence, the tests of significance for our coefficients of interest in our primary models were corrected for multiple hypothesis testing. In particular, we used a Bonferroni correction on all p-values and CI in our main analyses using an alpha of 5% adjusted to reflect that we run a separate model on each of 11 health conditions. Dataset construction was done in SAS System, Version 9.4 (SAS Institute, Cary, NC, USA) and all statistical analyses were done in Stata/IC 14.2 (StataCorp LP, College Station, TX, USA).

### Ethics statement

The current study involved retrospective analysis of existing, de-identified Medicare claims data and no new, primary data from human subjects was collected, so the study fell under one of the exemptions for secondary research under §46.104(d)(4) of the revised Common Rule and IRB approval was not required.

## Results

### Descriptive characteristics

Descriptive statistics for all Medicare beneficiaries age 65 and above who were observed between 2011 and 2016 are presented in Table 1, which compares demographic and geographic characteristics for people with HIV and people without diagnosed HIV. On average, beneficiaries living with HIV were first observed in our sample when they were slightly older than people without diagnosed HIV (median of 70 years versus 69 years) but were observed for fewer years (potentially reflecting higher mortality), so overall our sample of individuals with HIV was slightly younger. They are also more likely to be male (63% versus 45%), dually enrolled in Medicaid (43% versus 13%), and non-Hispanic Black (34% versus 10%) or Hispanic (13% versus 8%), and less likely to be non-Hispanic White (51% versus 80%) or non-Hispanic Asian (2% versus 3%). Finally, there were geographic differences, with beneficiaries with an HIV diagnosis less likely to reside in rural areas (8% versus 19%), more likely to live in the South (43% versus 37%) or the Northeast (27% versus 19%) and less likely to live in the Midwest (12% versus 22%) or West (19% versus 22%).

**Table 1:** Demographic and geographic characteristics, (Medicare beneficiaries aged 65 and over between 2011–2016).

	People with HIV			People without diagnosed HIV			Significance of difference		
Age	Median age first observed	Mean number of years observed	Median over pooled observations	Median age first observed	Mean number of years observed	Median over pooled observations			
	70	4	70	69	5	73	***	***	***
Male	63.1%			44.7%			***		
Medicaid	43.1%			13.4%			***		
White (non-Hispanic)	50.7%			79.6%			***		
Black (non-Hispanic)	34.4%			9.5%			***		
Hispanic	13.2%			7.5%			***		
Asian (non-Hispanic)	1.8%			3.4%			***		
Rural	8.0%			19.1%			***		
Northeast	26.6%			19.0%			***		
Midwest	12.1%			22.2%			***		
South	42.6%			37.3%			***		
West	18.7%			21.5%			***		
N	43,708			1,029,518					

Each individual appears in this table once, for most variables the value when they are first observed is used except for median age observed, which pools observations across all years of the data. Upper age is trimmed at 98 years to avoid the impact of outliers (who predominantly do not have HIV). Statistical significance of differences in medians and binary variables were calculated for each row using a chi-squared test, differences in significance of differences in means were calculated using a t-test. Significance levels are shown with *** p-value<0.001, ** p-value < 0.01, and * p-value < 0.05.

### Mortality hazard

**Fig 1:**
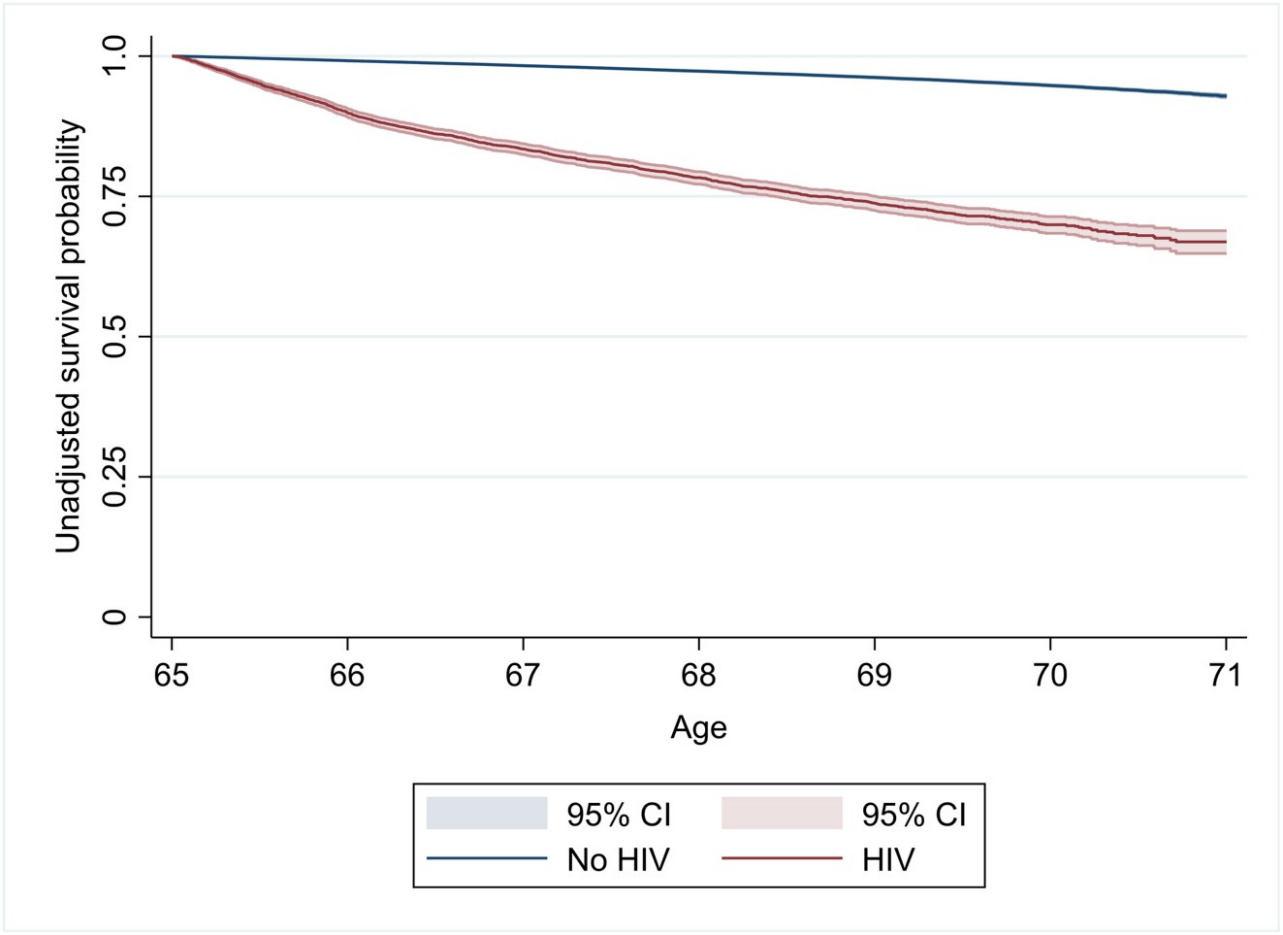
Kaplan-Meier plot of unadjusted survival by HIV status. Years since age 65.

Of the 321,999 individuals included in the survival analysis (including 6,927 people with HIV), 12,537 of them died (including 1,435 people with HIV) during 1,278,600 person-years of observation. In the Kaplan-Meier plot (Fig 1), unadjusted survival curves for people with HIV were significantly different than for older people without diagnosed HIV (the p-value for the log-rank test for the overall difference was 0.0000).

After adjusting for other demographic and geographic characteristics in the Cox proportional hazard model, an HIV diagnosis was associated with a hazard ratio (HR) of all-cause mortality that was approximately 3.6 times the HR of older adults without diagnosed HIV (column 1 of Table 2). The HR was even higher when we included an interaction with exposure time (years since age 65) to address non-proportional hazards (HR = 5.90), although this ratio decreased by approximately 1% for each year of exposure (column 2). Beneficiaries who are male, non-Hispanic Blacks, rural, or dually enrolled in Medicaid all had higher HRs in every specification of the model. The primary result—the mortality hazard was higher for people with HIV than for people without HIV—was robust across all specifications of the model.

**Table 2:** Cox proportional model, hazard ratios reported (Medicare beneficiaries observed at age 65 between 2011–2016).

	(1) Basic model	(2) Basic model + time-varying coefficients^a^	(3) Extended model (sex)	(4) Extended model (race/ethnicity)	(5) Extended model (Medicaid dual enrollee)	(6) Extended model (Rural)	(7) Extended model (original entitlement)^b^
HIV	3.59***	5.90***	3.81***	4.02***	5.84***	3.54***	2.21***
	[3.38, 3.83]	[5.33, 6.53]	[3.39, 4.29]	[3.69, 4.38]	[5.33, 6.45]	[3.31, 3.78]	[2.08, 2.36]
Male	1.66***	1.66***	1.66***	1.66***	1.66***	1.66***	1.60***
	[1.60, 1.72]	[1.60, 1.72]	[1.60, 1.72]	[1.60, 1.72]	[1.60, 1.72]	[1.60, 1.72]	[1.54, 1.66]
Black	1.13***	1.26***	1.26***	1.13***	1.13***	1.13***	1.02
	[1.07, 1.20]	[1.14, 1.39]	[1.14, 1.39]	[1.07, 1.20]	[1.07, 1.20]	[1.07, 1.20]	[0.97, 1.08]
Asian	0.39***	0.39***	0.39***	0.39***	0.39***	0.39***	0.47***
	[0.34, 0.45]	[0.34, 0.45]	[0.34, 0.45]	[0.34, 0.45]	[0.34, 0.45]	[0.34, 0.45]	[0.41, 0.54]
Hispanic	0.59***	0.59***	0.59***	0.59***	0.59***	0.59***	0.62***
	[0.55, 0.64]	[0.55, 0.64]	[0.55, 0.64]	[0.55, 0.64]	[0.55, 0.64]	[0.55, 0.64]	[0.57, 0.67]
Medicaid	3.57***	3.57***	3.57***	3.57***	3.57***	3.57***	2.62***
	[3.41, 3.73]	[3.41, 3.74]	[3.41, 3.74]	[3.41, 3.74]	[3.41, 3.74]	[3.41, 3.74]	[2.50, 2.76]
Rural	1.06*	1.06*	1.06*	1.06*	1.06*	1.06*	1.00
	[1.01, 1.11]	[1.01, 1.11]	[1.01, 1.11]	[1.01, 1.11]	[1.01, 1.11]	[1.01, 1.11]	[0.95, 1.05]
Interaction of HIV status with years since 65		0.999***					
		[0.999, 0.999]					
Interaction of HIV status with male sex			0.93				
			[0.81, 1.05]				
Interaction of HIV status with Black race				0.80***			
				[0.71, 0.91]			
Interaction of HIV status with Asian race				1.00			
				[0.51, 1.96]			
Interaction of HIV status with Hispanic ethnicity				0.94			
				[0.77, 1.14]			
Interaction of HIV status with Medicaid enrollment					0.52***		
					[0.46, 0.58]		
Interaction of HIV status with rural residence						1.20	
						[1.00, 1.45]	
Disability							2.71***
							[2.59, 2.83]
ESRD							8.84***
							[7.35, 10.63]
Disability and ESRD							10.45***
							[8.81, 12.40]
Log pseudolikelihood	-18,268,062	-18,254,350	-18,268,062	-18,268,054	-18,267,999	-18,268,060	-18,147,357

Includes robust standard errors clustered by beneficiary and year aged into Medicare fixed effects. The omitted group is non-Hispanic White, urban, non-Medicaid enrolled males. Individuals are weighted using probability weights to reflect differential selection probabilities based on HIV diagnosis. 95% confidence intervals are reported in parentheses. Significance levels are shown with *** p-value<0.001, ** p-value < 0.01, and * p-value < 0.05.

^a^ Includes year*exposure time and non-Hispanic black*exposure time interaction terms

^b^ The omitted group are individuals originally entitled to Medicare because of age.

In Columns 3 through 6 we added interaction terms between HIV diagnosis and various demographic and personal characteristics. The association between mortality hazard and HIV was not significantly different for men compared to women (Column 3), for either Hispanic or non-Hispanic Asian compared to non-Hispanic White beneficiaries (Column 4), or for rural compared to urban beneficiaries (Column 6). On the other hand, the association between HIV infection and all-cause mortality hazard was smaller for non-Hispanic Black beneficiaries than for non-Hispanic White beneficiaries (HR of 0.80). This is not a surprising result since non-Hispanic Black beneficiaries overall had a higher hazard of all-cause mortality, leaving less room for a differential effect of having HIV. A similar relationship applies to other findings presented in this analysis. For instance, the association between HIV infection and all-cause mortality hazard was smaller for dually-enrolled beneficiaries (OR = 0.52), while the mortality hazard of individuals dually enrolled in Medicaid, overall, was substantially higher. Finally, in Column 7, as a robustness check we added a control for whether the individual originally became eligible for Medicare based on age, end-stage renal disease, or disability (among our sample of people with HIV, 60% originally became eligible for Medicare based on age, 38% originally became eligible because of disability, and the remaining <2% originally became eligible because they had ESRD or ESRD and disability). This attenuated the coefficient on HIV diagnosis but it is still large and significant (HR = 2.2). Beneficiaries who originally became eligible because of a disability or end-stage renal disease have much a higher mortality hazard than those who originally qualified based on aging into the program.

### Prevalence

People with HIV had higher overall unadjusted rates for all health conditions compared to those without diagnosed HIV (Columns 1 through 3 in Table 3). The same calculations for subpopulations of interest including rural residents, those dually enrolled in Medicaid, minorities, and by sex can be found in S3 Table. People with HIV were, for example, almost 600% more likely to have COPD and over 300% more likely to have osteoporosis, hypertension, and heart disease compared to older people without diagnosed HIV. The difference is particularly striking for chronic hepatitis and end-stage liver disease, diseases which were more than 1,000% more common among older people with HIV compared to older people without diagnosed HIV.

**Table 3:** Prevalence of health outcomes, unadjusted means and odds ratios reported.

	Medicare beneficiaries at age 65 between 2011–2016, unadjusted			Coefficients from logistic regression model, odds ratios reported (Medicare beneficiaries aged 65 and above in 2016)^a^
	(1)	(2)	(3)	(4)
	HIV	No HIV	Percent difference	HIV
Depression	24.3%	6.2%	291.9%***	2.29*** [2.22, 2.35]
Chronic kidney disease	46.2%	18.9%	144.4%***	1.92*** [1.88, 1.97]
COPD	28.3%	4.1%	590.2%***	1.52*** [1.47, 1.57]
Osteoporosis	14.6%	3.4%	329.4%***	2.17*** [2.06, 2.29]
Colorectal cancer	3.8%	1.3%	192.3%***	1.85*** [1.70, 2.03]
Lung cancer	26.8%	7.7%	248.1%***	1.38*** [1.25, 1.53]
Hypertension	1.4%	0.3%	366.7%***	1.31*** [1.28, 1.33]
Ischemic heart disease	1.3%	0.3%	333.3%***	1.31*** [1.28, 1.34]
Diabetes	29.8%	10.4%	186.5%***	1.06*** [1.04, 1.09]
Chronic hepatitis	13.1%	0.2%	6450.0%***	12.70*** [11.86, 13.59]
End-stage liver disease	2.7%	0.1%	2600.0%***	4.51*** [4.08, 4.98]
N	8,277	330,955		1,024,823

Each individual appears in this table once; in columns one through three it is at age 65, in column 4 it is in 2016 (in which upper age is trimmed at 98 years to avoid the impact of outliers who predominantly do not have HIV). Significance of the differences between (1) and (2) were calculated for each row using a chi-squared test. Not shown are controls for age, race/ethnicity, sex, state, rural/urban residence, and dual enrollment in Medicaid. The omitted group is White, urban, non-Medicaid enrolled males between the age of 65 and 74. Individuals are weighted using probability weights to reflect differential selection probabilities based on HIV diagnosis. 95% confidence intervals are reported in parentheses. Significance levels are shown with *** p-value<0.001, ** p-value < 0.01, and * p-value < 0.05.

^a^ Each cell in (4) is a coefficient from a different model. Significance levels have been adjusted using the Bonferroni correction for multiple hypothesis testing to reflect 11 tests.

These patterns remained even after adjusting for individual characteristics in the logit models, although there is quite a range in magnitude of these relationships (Column 4 in Table 3 shows the coefficients on the HIV indicator variable for each of 11 separate regressions). The effect sizes are particularly striking for chronic hepatitis (the odds of having hepatitis were over 12 times as likely for older people with HIV, although baseline odds of hepatitis for people without diagnosed HIV were very low), and end-stage liver disease (the odds were over 4 times as likely for people with HIV). Looking at the other rows, compared to older people without HIV, older people with HIV had 2.3 times the odds of depression, 1.9 times the odds of chronic kidney disease, 1.5 times the odds of COPD, 2.2 times the odds of osteoporosis, 1.9 times the odds of colorectal cancer, 1.4 times the odds of lung cancer, 1.3 times the odds of hypertension, 1.3 times the odds of ischemic heart disease, and 1.1 times the odds of diabetes. As a robustness check, we ran these same models on other years to see whether the patterns changed over time, but the results were very consistent across the years (S2 Table).

The relationships between HIV infection and the odds of each health outcome for beneficiaries in rural areas was often smaller than that seen in urban areas, in particular for the odds of depression, chronic kidney disease, COPD, ischemic heart disease, and diabetes (Table 4). On the other hand, the increase in odds of having hepatitis was larger for people with HIV in rural areas than in urban areas. Medicaid dual enrollees, even without an HIV diagnosis, had higher odds of many conditions than non-dual enrollees without HIV, hence the additional increase in the OR for every condition (except for lung cancers) associated with having HIV was smaller for people who are dually enrolled in Medicaid than it was for those who were not dually enrolled. The story was more complex for minority beneficiaries compared to non-Hispanic White beneficiaries. The association between HIV infection and odds of COPD, lung cancer, hypertension, and ischemic heart disease was larger for minorities, but the association between HIV infection and odds of osteoporosis, colorectal cancer, end-stage liver disease, and hepatitis was smaller (although underlying hepatitis odds for minorities were quite high). Finally, in the sex comparison, the associations between HIV and the odds of having depression, chronic kidney disease, osteoporosis, hypertension, and diabetes were greater for men than for women (although women had higher rates of osteoporosis and depression overall), while the association was smaller for COPD, ischemic heart disease, and end-stage liver disease.

**Table 4:** Logit models of prevalence of health outcomes in 2016 for sub-groups of interest, odds ratios reported (Medicare beneficiaries aged 65 and older in 2016).

	(1)	(2)	(3)	(4)	(5)	(6)	(7)	(8)	(9)	(10)	(11)
	Depression	Chronic kidney disease	COPD	Osteoporosis	Colorectal cancer	Lung cancer	Hypertension	Ischemic heart disease	Diabetes	Hepatitis	End-stage liver disease
**Urban/Rural residence**											
HIV	2.30***	1.94***	1.56***	2.17***	1.86***	1.44***	1.32***	1.32***	1.07***	12.31***	4.45***
	[2.24, 2.38]	[1.89, 1.99]	[1.51, 1.62]	[2.06, 2.30]	[1.69, 2.04]	[1.29, 1.61]	[1.29, 1.34]	[1.28, 1.35]	[1.04, 1.10]	[11.48, 13.20]	[4.01, 4.93]
Rural resident	2.95	6.93**	0.41	1.82	1.48	1.33	0.03**	0.12*	0.17*	1.18	0.98
	[0.67, 12.95]	[2.09, 22.96]	[0.06, 2.97]	[0.23, 14.38]	[0.84, 2.62]	[0.70, 2.53]	[0.00, 0.22]	[0.02, 0.87]	[0.04, 0.69]	[0.28, 4.95]	[0.51, 1.92]
Interaction of HIV status and rural residence	0.89*	0.89*	0.85**	0.92	1.04	0.76	0.90**	0.91*	0.87**	1.56**	1.06
	[0.80, 0.99]	[0.81, 0.97]	[0.76, 0.95]	[0.75, 1.13]	[0.77, 1.41]	[0.52, 1.10]	[0.83, 0.97]	[0.83, 0.99]	[0.79, 0.95]	[1.20, 2.03]	[0.74, 1.51]
**Dual enrollment in Medicaid**											
HIV	2.55***	2.16***	1.54***	2.45***	1.90***	1.38***	1.32***	1.39***	1.22***	16.90***	6.03***
	[2.45, 2.65]	[2.10, 2.23]	[1.47, 1.61]	[2.27, 2.63]	[1.70, 2.13]	[1.20, 1.58]	[1.29, 1.36]	[1.34, 1.43]	[1.18, 1.26]	[15.59, 18.33]	[5.35, 6.79]
Medicaid enrollee	2.93***	1.51	5.09***	0.76	1.20	4.62*	1.23	1.75*	1.75***	8.22*	4.34
	[1.85, 4.64]	[0.93, 2.47]	[3.25, 7.97]	[0.31, 1.89]	[0.17, 8.62]	[1.42, 15.08]	[0.84, 1.82]	[1.13, 2.71]	[1.13, 2.70]	[1.08, 62.31]	[0.59, 31.77]
Interaction of HIV status and Medicaid	0.76***	0.77***	0.87***	0.72***	0.81*	0.85	0.95*	0.88***	0.82***	0.58***	0.55***
	[0.71, 0.80]	[0.74, 0.81]	[0.81, 0.93]	[0.64, 0.80]	[0.70, 0.97]	[0.69, 1.05]	[0.91, 0.99]	[0.83, 0.92]	[0.78, 0.86]	[0.51, 0.65]	[0.46, 0.67]
**Racial and ethnic minorities/non-Hispanic White**											
HIV	2.37***	1.99***	1.34****	2.31***	2.12***	1.26**	1.18***	1.30***	1.10***	15.48***	5.11***
	[2.28, 2.46]	[1.93, 2.06]	[1.28, 1.40]	[2.15, 2.48]	[1.90, 2.37]	[1.10, 1.44]	[1.15, 1.21]	[1.26, 1.34]	[1.06, 1.14]	[14.07, 17.03]	[4.52, 5.77]
Racial and ethnic minority	1.94*	1.08	0.75	0.33***	2.57	1.24	0.05**	1.31	0.42	3.99***	3.68
	[1.14, 3.31]	[0.60, 1.92]	[0.35, 1.58]	[0.22, 0.49]	[0.62, 10.72]	[0.17, 8.85]	[0.01, 0.40]	[0.81, 2.13]	[0.10, 1.77]	[2.01, 7.93]	[0.51, 26.47]
Interaction of HIV status and minority	0.98	1.05	1.45***	0.64***	0.77**	1.30*	1.26***	1.07**	1.02	0.78***	0.82*
	[0.92, 1.04]	[1.00, 1.10]	[1.35, 1.55]	[0.57, 0.71]	[0.64, 0.92]	[1.05, 1.61]	[1.21, 1.31]	[1.02, 1.13]	[0.98, 1.07]	[0.69, 0.88]	[0.68, 0.99]
**Sex**											
HIV	1.86***	1.84***	1.73***	1.73***	1.59***	1.51***	1.24***	1.42***	1.05*	12.06***	5.47***
	[1.78, 1.95]	[1.76, 1.92]	[1.63, 1.83]	[1.62, 1.85]	[1.33, 1.90]	[1.25, 1.82]	[1.20, 1.29]	[1.35, 1.48]	[1.00, 1.09]	[10.78, 13.48]	[4.59, 6.51]
Male sex	0.53***	0.94	1.07	0.08***	1.94*	0.65	0.04**	1.57***	0.15	0.74	1.04
	[0.40, 0.70]	[0.77, 1.16]	[0.83, 1.37]	[0.04, 0.16]	[1.07, 3.51]	[0.23, 1.80]	[0.01, 0.31]	[1.32, 1.86]	[0.02, 1.08]	[0.10, 5.51]	[0.24, 4.41]
Interaction of HIV status and male sex	1.29***	1.07**	0.81***	1.72***	1.19	0.86	1.05*	0.94*	1.07*	0.99	0.73**
	[1.22, 1.37]	[1.02, 1.13]	[0.75, 0.87]	[1.53, 1.92]	[0.97, 1.47]	[0.69, 1.08]	[1.01, 1.10]	[0.89, 0.99]	[1.01, 1.12]	[0.87, 1.14]	[0.59, 0.90]

Each cell is a coefficient from a different model. Not shown are controls for age, race/ethnicity, sex, state, rural/urban residence, and dual enrollment in Medicaid. The omitted group is White, urban, non-Medicaid enrolled males between the age of 65 and 74. Individuals are weighted using probability weights to reflect differential selection probabilities based on HIV diagnosis. 95% confidence intervals are reported in parentheses. Significance levels are shown with *** p-value<0.001, ** p-value < 0.01, and * p-value < 0.05.

### Incidence

**Fig 2:**
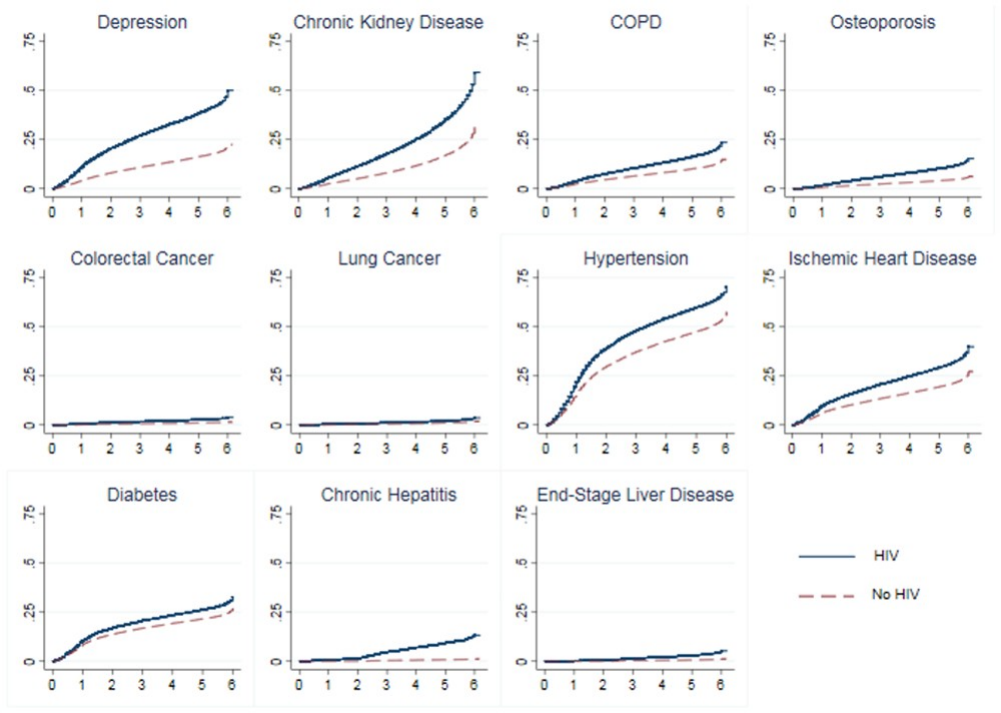
Cumulative incidence functions by HIV status. The y-axis is probability of diagnosis of each comorbidity and the x-axis is years since age 65.

Having an HIV diagnosis was associated with an increased subhazard of every condition in our analysis (Table 5). Subhazards are not straightforward to interpret on their own, so for this type of analysis, it is more useful to look graphically at cumulative incidence functions (Fig 2), which plot the probability of observing a particular event (e.g., a diagnosis of depression) by a given time, and is a function of the competing risk of mortality. These graphs show both how the difference in cumulative incidence between people with and without HIV changes as they age, but also gives a sense of the level of each condition in the population overall. We can see, for instance, that the cumulative incidence for diagnosis of depression was nearly 50% by age 72 for people with HIV, whereas it was closer to 20% for people without a diagnosis of HIV. There’s a similarly large gap for chronic kidney disease, where people with HIV have a cumulative incidence by age 72 of nearly 60%, while people without HIV have a cumulative incidence of approximately 30%. The gaps for the other conditions analyzed are smaller but still significant. For instance, the probability of a diagnosis of COPD for people with HIV by age 72 was nearly 25%, compared to approximately 15% for people without diagnosed HIV. We can also observe that although the differences in cumulative incidence were different for people with and without HIV for colorectal cancer, lung cancer, and end-stage liver disease, these were much less common conditions compared to some of the others analyzed such as hypertension or chronic kidney disease. The overall pattern of results of the subgroup analyses are similar to those observed in the prevalence models. However, results were often more strongly significant in these incidence models.

**Table 5:** Competing risk models of the hazard of developing conditions over time, subhazard ratios reported (Medicare beneficiaries who are observed at age 65 between 2011 and 2016).

	(1)	(2)	(3)	(4)	(5)	(6)	(7)	(8)	(9)	(10)	(11)
	Depression	Chronic kidney disease	COPD	Osteoporosis	Colorectal cancer	Lung cancer	Hypertension	Ischemic heart disease	Diabetes	Hepatitis	End-stage liver disease
**Basic model**											
HIV	2.73***	2.31***	1.67***	2.61***	2.59***	1.97***	1.41***	1.60***	1.25***	11.50***	4.41***
	[2.56, 2.89]	[2.17, 2.44]	[1.55, 1.80]	[2.26, 2.95]	[2.04, 3.14]	[1.52, 2.43]	[1.35, 1.48]	[1.51, 1.70]	[1.18, 1.32]	[10.15, 12.86]	[5.59, 5.24]
**Extended model—Urban/rural residence**											
HIV	2.98***	2.58***	1.80***	2.71***	2.66***	2.08***	1.51***	1.69***	1.34***	12.62***	4.86***
	[2.85, 3.13]	[1.47, 2.70]	[1.70, 1.91]	[2.44, 3.01]	[2.24, 3.15]	[1.73, 2.50]	[1.45, 1.57]	[1.62, 1.78]	[1.28, 1.41]	[11.54, 13.81]	[4.21, 5.62]
Interaction of HIV status and rural residence	0.80**	0.91	0.81*	1.05	1.24	0.93	0.95	0.91	0.86	1.11	0.64
	[0.69, 0.94]	[0.78, 1.06]	[0.68, 0.98]	[0.75, 1.46]	[0.80, 1.94]	[0.53, 1.62]	[0.84, 1.07]	[0.77, 1.06]	[0.74, 1.01]	[0.83, 1.48]	[0.38, 1.09]
**Extended model—Dual enrollment in Medicaid**											
HIV	4.46***	3.87***	2.75***	4.36***	3.51***	2.23***	1.90***	2.27***	1.97***	25.18***	9.73***
	[4.16, 4.79]	[3.62, 4.13]	[2.50, 3.03]	[3.72, 5.11]	[2.75, 4.47]	[1.65, 3.02]	[1.80, 1.32]	[2.12, 2.44]	[1.84, 2.11]	[22.37, 28.34]	[7.96, 11.90]
Interaction of HIV status and Medicaid enrollment	0.56***	0.56***	0.56***	0.52***	0.68*	0.90	0.70***	0.65***	0.58***	0.42***	0.38***
	[0.51, 0.61]	[0.51, 0.61]	[0.50, 0.63]	[0.43, 0.64]	[0.50, 0.94]	[0.63, 1.30]	[0.66, 0.76]	[0.59, 0.70]	[0.53, 0.63]	[0.38, 0.49]	[0.30, 0.49]
**Extended model—Racial and ethnic minority/non-Hispanic White**											
HIV	3.05***	2.85***	1.67***	3.22***	3.88***	2.00***	1.40***	1.67***	1.42***	16.06***	5.56***
	[2.86, 3.25]	[2.67, 3.05]	[1.54, 1.81]	[2.80, 3.72]	[3.17, 4.75]	[1.56, 2.56]	[1.32, 1.47]	[1.57, 1.79]	[1.33, 1.53]	[14.14, 18.24]	[4.59, 6.73]
Interaction of HIV status and Black race	0.87**	0.84***	1.04	0.62***	0.48***	1.03	1.09*	1.00	0.86**	0.71***	0.73*
	[0.79, 0.95]	[0.77, 0.99]	[0.93, 1.17]	[0.50, 0.78]	[0.34, 0.67]	[0.73, 1.46]	[1.01, 1.18]	[0.91, 1.10]	[0.79, 0.95]	[0.61, 0.83]	[0.55, 0.97]
Interaction of HIV status and Asian race	1.60	1.06	1.35	1.06	0.00	3.99***	1.24	1.43	1.31	0.21***	2.18
	[0.99, 2.63]	[0.70, 1.60]	[0.66, 2.76]	[0.48, 2.35]	[0.00, 0.00]	[1.21, 13.17]	[0.86, 1.80]	[0.97, 2.12]	[0.89, 1.93]	[0.11, 0.42]	[0.84, 4.69]
Interaction of HIV status and Hispanic ethnicity	1.11	0.85***	2.75***	1.00	0.65	1.08	1.29***	1.00	1.01	0.95	0.80
	[0.97, 1.27]	[0.74, 0.97]	[1.23, 1.76]	[0.77, 1.30]	[0.39, 1.09]	[0.50, 2.31]	[1.16, 1.44]	[0.87, 1.16]	[0.88, 1.15]	[0.75, 1.20]	[0.57, 1.14]
**Extended model—Sex**											
HIV	2.24***	2.54***	2.00***	1.78***	1.70**	2.40***	1.54***	2.18***	1.48***	10.97***	4.72***
	[2.07, 2.42]	[2.34, 2.76]	[1.77, 2.16]	[1.55, 2.04]	[1.18, 2.45]	[1.77, 3.25]	[1.44, 1.65]	[2.00, 2.67]	[1.37, 1.60]	[9.55, 12.60]	[3.66, 6.08]
Interaction of HIV status and male sex	1.50***	1.01	0.87**	2.94***	1.82**	0.81	0.98	0.72***	0.86**	1.21*	1.00
	[1.37, 1.65]	[0.92, 1.11]	[0.77, 0.98]	[2.43, 3.55]	[1.23, 2.69]	[0.57, 1.16]	[0.90, 1.05]	[0.65, 0.79]	[0.79, 0.94]	[1.04, 1.41]	[0.76, 1.33]

Each column is from a different model. Not shown are controls for race/ethnicity, sex, rural/urban residence, and dual enrollment in Medicaid. The omitted category is non-Hispanic White, female, non-dually enrolled, urban beneficiaries without diagnosed HIV. Individuals are weighted using probability weights to reflect differential selection probabilities based on HIV diagnosis. Robust standard errors are clustered by beneficiary. 95% confidence intervals are reported in parentheses. Significance levels are shown with *** p-value<0.001, ** p-value < 0.01, and * p-value < 0.05. Confidence intervals and p-values for the basic model have been adjusted using the Bonferroni correction for multiple hypothesis testing.

## Discussion

This paper analyzed a large, nationally representative sample of Medicare beneficiaries aged 65 and older from 2011 to 2016 to compare the health and survival status of older people with HIV to an older US population not living with HIV. Prior research has found that older people with HIV have high adherence to treatment which results in high levels of viral suppression [25, 26]. Adherence to treatment and viral suppression are both associated with better health outcomes [51]. Nonetheless, we found that older people with HIV have a higher overall hazard of mortality as well as a higher odds of having depression, chronic kidney disease, COPD, osteoporosis, colorectal cancer, lung cancer, hypertension, ischemic heart disease, diabetes, chronic hepatitis, and end-stage liver disease compared to those without HIV, even after adjusting for demographic characteristics. Some of these differences were quite large in magnitude, particularly for hepatitis and end-stage liver disease. Finally, we found that the incidence of diagnosis over time of every condition analyzed is higher for people with HIV, after accounting for the competing and differential risk of mortality.

Our results differed significantly by population subgroup. We found that older people with HIV are more likely to be from potentially underserved populations, including minorities (49% of older people with HIV) and dual Medicaid enrollees (43% of older people with HIV). We also found that minorities and dual Medicaid enrollees overall had higher hazards of mortality as well as higher odds and incidence of many conditions (even without an HIV diagnosis), and that an HIV diagnosis is associated with even larger disparities.

This project benefited from using a large, population-representative, longitudinal sample, which allowed us to observe individuals over multiple years and to demonstrate the need to consider selective mortality in future research looking at the aging population of people with HIV. There were, however, some limitations due to data constraints that future work should try to address. The first and foremost is that we cannot observe what occurred among individuals in our sample when they were younger, which is when most were infected or diagnosed, and we do not know their initial date of diagnosis. We also do not have certain types of clinical information such as their use of ARTs or CD4 count or viral load. This means we cannot disentangle whether these observed differences in mortality and health are due to the impact of HIV itself, the effect of prolonged use of ARTs, or other individual characteristics that may be associated with HIV that we do not observe. For instance, people with HIV have higher rates of tobacco use, and tobacco use is associated with a higher risk of having a number of the health outcomes studied in this paper as well as higher mortality [52]. It is also possible that people with HIV have higher diagnosis rates for conditions because of more frequent contact with the medical system, particularly for conditions that are known to commonly co-occur with HIV. Hence, our findings in this paper should not be considered direct estimates of the effect of the HIV virus on health status, rather we are estimating the health status of individuals with HIV whose health status and mortality hazard may vary from those without HIV for multiple reasons.

Concerning the analyses about chronic hepatitis, due to limitations in what is reported in claims data, our analyses focused on chronic hepatitis generally, although people with HIV are known to be at higher risk for hepatitis C in particular [53]. The Centers for Disease Control (CDC) guidelines also specify that everyone born between 1945 and 1965 (a range capturing most of our sample) be tested for hepatitis C at least once, as they are at higher risk for this disease [54]. Therefore, while our results for hepatitis are not unexpected, the extremely high rates of co-occurrence of HIV and hepatitis in older people with HIV reaffirm the need for screening, particularly for hepatitis C. Hepatitis C infection can result in significant health complications, but is now curable.

While our data do not allow us to analyze the underlying factors contributing to these outcomes, our results nonetheless raise important questions regarding the role of social determinants of health for people with HIV. People with HIV are disproportionately more likely to be from underserved and vulnerable groups, including men who have sex with men, transgender people, people of color, and those with lower socioeconomic status [55], and these disparities may affect HIV prevention; testing; and access to care, treatment, and support services, leading to potentially worse health outcomes and poorer survival. Stigma and social isolation are widely documented challenges for many people with HIV, particularly for men who have sex with men and transgender individuals, and older people with HIV tend to be particularly isolated with limited social networks [18, 56–58]. As these individuals age and develop more chronic conditions and increasingly complex care needs, they often have fewer resources and family members to rely on [59]. Thus, older people with HIV may be more dependent on formal care but may also face significant barriers to access, including having fewer personal resources relative to their healthcare needs and stigma. Compounding these challenges, older people with HIV also have higher rates of mental health conditions, including depression, as well as higher rates of substance use and homelessness, all of which can contribute to challenges in accessing and adhering to care [18].

In addition to the personal health and social implications for older people with HIV, these results have important implications for community partners and programs as they plan for an aging population, including Medicare, Medicaid, and the Health Resources and Services Administration’s Ryan White HIV/AIDS Program, which provides a comprehensive system of care to low-income people with HIV in the United States, including over 230,000 people with HIV aged 50 and older (approximately half of all people with HIV aged 50 years and older in the United States) [1, 60].

These results are also important for clinicians caring for people with HIV, who are most likely to be treated by primary care physicians or infectious disease specialists [61] and therefore may not have the same level of experience with caring for older adults as specialists in gerontology or geriatrics. This suggests a need for training of medical professionals on the intersecting issues of aging and HIV, particularly in how to deal with multi-morbidity, polypharmacy, and the need for personalized care and maximizing functional capacity. It also points to the need for increased coordination and integration of services, including HIV services and geriatric services, as well as an increased focus by providers on prevention, screening, and treatment for other conditions for which older people with HIV are at higher risk. The Ryan White HIV/AIDS Program’s AIDS Education and Training Centers are one important mechanism by which medical professionals can share information with each other on lessons learned in treatment and coordinating care for older people with HIV.

Understanding the degree to which individuals living with HIV may have different healthcare needs, regardless of the underlying causes of differences in health status for these individuals, is of policy interest. Future research should study the underlying reasons for differences in health status, as well as at what age these differences begin to appear, to better understand particular clinical interventions that may be applicable to treating comorbidities in this population.

While much attention is currently being devoted to ending the HIV epidemic, which is an important initiative, a cure does not yet exist for those currently living with HIV, and so it is also important that efforts continue to be made to improve health outcomes and quality of life for this population. Our findings of markedly higher odds of comorbid conditions, in combination with previous work linking comorbidities with decreased quality of life among older people with HIV [62, 63], highlights the need for further efforts to improve care for these individuals.

## Supporting information

S1 Table Alternate specifications of the Cox proportional model, hazard ratios reported (Medicare beneficiaries). (DOCX)

S2 Table Logit models of health outcomes, additional years, odds ratios reported (Medicare beneficiaries aged 65 and above in 2011, 2013, or 2016, depending on row). (DOCX)

S3 Table Health outcomes by population subgroup (Medicare beneficiaries aged 65 and over between 2011–2016). (DOCX)
