# Supplementary material for: Assessing the health status and mortality of older people over 65 with HIV
Source: PLoS One. 2020 Nov 5;15(11):e0241833. doi: 10.1371/journal.pone.0241833 (PMC7644038; doi:10.1371/journal.pone.0241833)
Supplement: S1 Table — (DOCX) [file pone.0241833.s001.docx]

|  | (1)  Basic model (begin at age 65) | (2)  Older cohort (begin at age 75) | (3)  All ages 65 and older with addition of controls for age^a^ | (4)  Basic model plus individuals whose HIV status changes | (5)  Basic model plus controls for other conditions^b^ | (6)  Basic model plus CMS-HCC risk score^c^ | (7)  Inverse probability weighted model^d^ |
| --- | --- | --- | --- | --- | --- | --- | --- |
| HIV | 3.59*** | 3.07*** | 4.04*** | 3.26*** | 2.02*** | 1.45*** | 4.19*** |
|  | [3.38, 3.83] | [2.86, 3.30] | [3.95, 4.13] | [3.08, 3.46] | [1.86, 2.21] | [1.20, 1.75] | [3.71, 4.73] |
| Male sex | 1.66*** | 1.54*** | 1.50*** | 1.66*** | 1.49*** | 1.60*** | 1.78*** |
|  | [1.60, 1.72] | [1.48, 1.59] | [1.48, 1.52] | [1.60, 1.72] | [1.43, 1.55] | [1.54, 1.68] | [1.68, 1.87] |
| Black race | 1.13*** | 1.04 | 0.93*** | 1.13*** | 1.13*** | 0.91* | 1.17*** |
|  | [1.07, 1.20] | [0.98, 1.10] | [0.91, 0.95] | [1.07, 1.20] | [1.07, 1.20] | [0.84, 0.98] | [1.08, 1.26] |
| Asian race | 0.39*** | 0.40*** | 0.44*** | 0.39*** | 0.54*** | 0.45*** | 0.47*** |
|  | [0.34, 0.45] | [0.35, 0.44] | [0.42, 0.46] | [0.34, 0.45] | [0.47, 0.62] | [0.39, 0.52] | [0.39, 0.58] |
| Hispanic ethnicity | 0.59*** | 0.60*** | 0.60*** | 0.59*** | 0.73*** | 0.57*** | 0.69*** |
|  | [0.55, 0.64] | [0.56, 0.64] | [0.58, 0.61] | [0.55, 0.64] | [0.68, 0.79] | [0.51, 0.62] | [0.61, 0.78] |
| Medicaid enrollee | 3.57*** | 2.53*** | 2.38*** | 3.57*** | 2.04*** | 2.12*** | 3.57*** |
|  | [3.41, 3.73] | [2.42, 264] | [2.35, 2.41] | [3.41, 3.73] | [1.93, 2.15] | [2.01, 2.24] | [3.27, 3.89] |
| Rural residence | 1.06* | 1.07** | 1.07*** | 1.06* | 1.01 | 1.11*** | 1.05 |
|  | [1.01, 1.11] | [1.03, 1.12] | [1.05, 1.08] | [1.01, 1.11] | [0.96, 1.06] | [1.05, 1.16] | [0.98, 1.13] |

Includes robust standard errors clustered by beneficiary. Year aged into Medicare fixed effects are included but not shown. The omitted group is non-Hispanic White, urban, non-Medicaid enrolled males. Individuals are weighted in columns (1) through (6) using probability weights to reflect differential selection probabilities based on HIV diagnosis. 95% confidence intervals are reported in parentheses. Significance levels are shown with *** p-value<0.001, ** p-value < 0.01, and * p-value < 0.05.

^a^ Minimum age observed is included linearly.

^b^ Includes binary indicators for hepatitis, liver disease, depression, chronic kidney disease, COPD, osteoporosis, colorectal cancer, lung cancer, hypertension, ischemic heart disease, and diabetes are included.

^c^ Includes the HCC risk score calculated by CMS, which includes a number of chronic conditions, including those we are interested in, as well as HIV diagnosis and demographic characteristics.

^d^ We constructed a stabilized weight by predicting HIV diagnosis with baseline demographic characteristics, geographic characteristics, and presence of each of the comorbid conditions included in column (5).
