# Supplementary material for: Assessing the health status and mortality of older people over 65 with HIV
Source: PLoS One. 2020 Nov 5;15(11):e0241833. doi: 10.1371/journal.pone.0241833 (PMC7644038; doi:10.1371/journal.pone.0241833)
Supplement: S2 Table — (DOCX) [file pone.0241833.s002.docx]

|  | (1) | (2) | (3) | (4) | (5) | (6) | (7) | (8) | (9) | (10) | (11) |
| --- | --- | --- | --- | --- | --- | --- | --- | --- | --- | --- | --- |
|  | Depression | Chronic kidney disease | COPD | Osteoporosis | Colorectal cancer | Lung cancer | Hypertension | Ischemic heart disease | Diabetes | Chronic hepatitis | End-stage liver disease |
| **2011** | | | | | | | | | | | |
| HIV | 2.74*** | 2.99*** | 2.01*** | 1.94*** | 2.01*** | 2.45*** | 1.41*** | 1.56*** | 1.28*** | 18.69*** | 6.77*** |
|  | [2.56, 2.92] | [2.81, 3.15] | [1.87, 2.15] | [1.74, 2.18] | [1.68, 2.42] | [2.02, 2.99] | [1.35, 1.48] | [1.48, 1.64] | [1.21, 1.35] | [15.80, 22.10] | [5.29, 8.67] |
| **2013** | | | | | | | | | | | |
| HIV | 2.68*** | 2.81*** | 1.99*** | 2.08*** | 2.04*** | 2.73*** | 1.44*** | 1.60*** | 1/26*** | 16.37*** | 6.82*** |
|  | [2.57, 2.79] | [2.71, 2.91] | [1.91, 2.08] | [1.94, 2.25] | [1.81, 2.30] | [2.42, 3.09] | [1.40, 1.48] | [1.55, 1.65] | [1.22, 1.31] | [14.83, 18.08] | [5.92, 7.86] |
| **Main model – 2016** | | | | | | | | | | | |
| HIV | 2.29*** | 1.92*** | 1.52*** | 2.17*** | 1.85*** | 1.38*** | 1.31*** | 1.31*** | 1.06*** | 12.70*** | 4.51*** |
|  | [2.22, 2.35] | [1.88, 1.97] | [1.47, 1.57] | [2.06, 2.29] | [1.70, 2.03] | [1.25, 1.53] | [1.28, 1.33] | [1.28, 1.34] | [1.04, 1.09] | [11.86, 13.59] | [4.08, 4.98] |
| **2016 – restricting model to individuals in the sample at age 65 (therefore included in the mortality and incidence models)** | | | | | | | | | | | |
| HIV | 2.61*** | 2.03*** | 1.57*** | 2.75*** | 2.04*** | 1.49*** | 1.36*** | 1.36*** | 1.08*** | 11.45*** | 4.15*** |
|  | [2.49, 2.72] | [1.95, 2.10] | [1.48, 1.65] | [2.51, 3.01] | [1.75, 2.38] | [1.23, 1.77] | [1.32, 1.40] | [1.31, 1.41] | [1.04, 1.12] | [10.45, 12.55] | [3.57, 4.82] |

Each cell is a coefficient from a different model. Not shown are controls for age, race/ethnicity, sex, state, rural/urban residence, and dual enrollment in Medicaid. Upper age is trimmed at 98 years to avoid the impact of outliers (who predominantly do not have HIV). The omitted group is White, urban, non-Medicaid enrolled males between the age of 65 and 74. Individuals are weighted using probability weights to reflect differential selection probabilities based on HIV diagnosis. 95% confidence intervals are reported in parentheses. Significance levels are shown with *** p-value<0.001, ** p-value < 0.01, and * p-value < 0.05. Confidence intervals and p-values for the main model have been adjusted using the Bonferroni correction for multiple hypothesis testing.
