# Supplementary material for: Assessing the health status and mortality of older people over 65 with HIV
Source: PLoS One. 2020 Nov 5;15(11):e0241833. doi: 10.1371/journal.pone.0241833 (PMC7644038; doi:10.1371/journal.pone.0241833)
Supplement: S3 Table — (DOCX) [file pone.0241833.s003.docx]

| **Panel A** | | | | | | | | | | |
| --- | --- | --- | --- | --- | --- | --- | --- | --- | --- | --- |
|  | HIV | No HIV | HIV | | No HIV | | HIV | | No HIV | |
|  | Full sample | Full sample | Rural | Urban | Rural | Urban | Medicaid | Non-Medicaid | Medicaid | Non-Medicaid |
| Depression | 24.3% | 6.2% | 27.1% | 24.1% | 7.3% | 5.9% | 27.7% | 19.3% | 15.9% | 4.8% |
| Hypertension | 46.2% | 18.9% | 49.2% | 45.9% | 22.8% | 18.1% | 50.5% | 39.6% | 37.6% | 16.3% |
| Chronic kidney disease | 28.3% | 4.1% | 30.0% | 28.4% | 4.4% | 4.0% | 31.1% | 24.0% | 11.4% | 3.1% |
| COPD | 14.6% | 3.4% | 19.1% | 14.2% | 4.9% | 3.0% | 17.7% | 9.9% | 11.1% | 2.3% |
| Osteoporosis | 3.8% | 1.3% | 4.6% | 3.8% | 1.4% | 1.3% | 4.3% | 3.1% | 3.0% | 1.1% |
| Heart disease | 26.8% | 7.7% | 30.0% | 26.5% | 9.6% | 7.3% | 28.9% | 23.5% | 16.9% | 6.4% |
| Colorectal cancer | 1.4% | 0.3% | 1.6% | 1.3% | 0.4% | 0.3% | 1.5% | 1.2% | 0.7% | 0.3% |
| Lung cancer | 1.3% | 0.3% | 1.4% | 1.3% | 0.4% | 0.3% | 1.6% | 0.9% | 0.6% | 0.2% |
| Diabetes | 29.8% | 10.4% | 30.4% | 29.8% | 12.4% | 9.9% | 33.3% | 24.5% | 24.3% | 8.4% |
| Chronic hepatitis | 13.1% | 0.2% | 6.5% | 13.6% | 0.1% | 0.2% | 16.0% | 8.8% | 0.8% | 0.1% |
| End-stage liver disease | 2.7% | 0.1% | 3.3% | 2.7% | 0.1% | 0.1% | 3.0% | 2.4% | 0.4% | 0.1% |
| N | 8,277 | 330,955 | 634 | 7,643 | 61,856 | 269,017 | 4,968 | 3,309 | 41,344 | 289,529 |
| **Panel B** | | | | | | | | | | |
|  | HIV | | No HIV | | HIV | | No HIV | |  |  |
|  | Racial and ethnic minority | Non-Hispanic White | Racial and ethnic minority | Non-Hispanic White | Men | Women | Men | Women |  |  |
| Depression | 21.2% | 28.5% | 5.0% | 6.6% | 22.6% | 28.9% | 4.1% | 8.1% |  |  |
| Hypertension | 51.4% | 39.2% | 23.1% | 17.8% | 43.7% | 52.6% | 18.4% | 19.4% |  |  |
| Chronic kidney disease | 32.3% | 22.9% | 6.4% | 3.4% | 28.2% | 28.5% | 4.5% | 3.7% |  |  |
| COPD | 14.0% | 15.4% | 3.0% | 3.5% | 13.9% | 16.4% | 3.3% | 3.4% |  |  |
| Osteoporosis | 2.8% | 5.2% | 1.2% | 1.4% | 2.7% | 6.8% | 0.3% | 2.3% |  |  |
| Heart disease | 26.6% | 27.0% | 8.1% | 7.6% | 27.1% | 25.8% | 9.8% | 5.9% |  |  |
| Colorectal cancer | 1.1% | 1.7% | 0.4% | 0.3% | 1.6% | 0.8% | 0.3% | 0.3% |  |  |
| Lung cancer | 1.2% | 1.5% | 0.2% | 0.3% | 1.3% | 1.4% | 0.3% | 0.3% |  |  |
| Diabetes | 34.8% | 23.3% | 15.0% | 9.1% | 27.6% | 35.6% | 10.5% | 10.3% |  |  |
| Chronic hepatitis | 16.4% | 8.6% | 0.4% | 0.1% | 13.6% | 11.8% | 0.2% | 1.4% |  |  |
| End-stage liver disease | 2.4% | 3.2% | 0.1% | 0.1% | 2.9% | 2.3% | 0.1% | 0.1% |  |  |
| N | 4,730 | 3,547 | 73,565 | 257,308 | 6,020 | 2,257 | 174,454 | 156,419 |  |  |

Each individual appears in this table once when they are first observed
